# Intestinal Behçet disease associated with myelodysplastic syndrome accompanying trisomy 8 successfully treated with abdominal surgery followed by hematopoietic stem cell transplantation: A case report

**DOI:** 10.1097/MD.0000000000017979

**Published:** 2019-11-15

**Authors:** Tomoyuki Asano, Shuzo Sato, Makiko Yashiro Furuya, Hiroshi Takahashi, Akiko Shichishima-Nakamura, Hiroshi Ohkawara, Tatsuo Fujiwara, Naohiko Gunji, Choichiro Hashimoto, Tomoyuki Momma, Motonobu Saito, Hiroshi Nakano, Guy Watanabe, Jumpei Temmoku, Yuya Fujita, Naoki Matsuoka, Hiroko Kobayashi, Hiroshi Watanabe, Mariko Mouri, Fumi Mashiyama, Hiroko Sakuma, Hiromasa Ohira, Masaaki Mori, Takayuki Ikezoe, Kiyoshi Migita

**Affiliations:** a Department of Rheumatology; b Department of Hematology; c Department of Gastroenterology; d Department of Gastrointestinal Tract Surgery, Fukushima Medical University School of Medicine, Fukushima; e Department of Pediatrics, Graduate School of Medicine, The University of Tokyo; f Department of Lifetime Clinical Immunology, Graduate School of Medical and Dental Sciences, Tokyo Medical and Dental University, Tokyo; g Department of Pediatrics, Hoshi General Hospital, Koriyama, Japan.

**Keywords:** hematopoietic stem cell transplantation, ileocecal resection, intestinal Behçet disease, myelodysplastic syndrome, peripheral blood stem cell transplantation, trisomy 8

## Abstract

**Rationale:**

Intestinal Behçet disease (BD) with myelodysplastic syndrome (MDS) is a rare condition that is resistant to various immunosuppressive therapies. Several cases in which hematopoietic stem cell transplantation (HSCT) was effective for intestinal BD with MDS accompanying trisomy 8 have been reported.

**Patient concerns:**

We report an 18-year-old female with a 7-year history of BD. Colonoscopy demonstrated a huge ulcer in the cecum. Chromosomal examination revealed a karyotype of trisomy 8 in 87% of cells. Bone marrow examination revealed dysplastic cells in multilineages.

**Diagnoses:**

A diagnosis of intestinal BD associated with MDS accompanying trisomy 8 was made.

**Interventions:**

The patient underwent ileocecal resection due to microperforations of ileocecal ulcers; she then underwent allogeneic peripheral blood stem cell transplantation (PBSCT) with her mother as a donor.

**Outcomes:**

After the PBSCT, the patient's symptoms due to BD (fever, oral aphthae, abdominal pain, and genital ulcers) completely disappeared, with no severe adverse events.

**Lessons:**

The present case demonstrates that HSCT including PBSCT might be an effective new therapeutic option for refractory intestinal BD with MDS when immunosuppressive therapy has achieved insufficient efficacy.

## 1 Introduction

Behçet disease (BD) is an inflammatory disease of unknown origin, characterized by recurrent oral aphthae, genital ulcers, uveitis, and skin lesions. Gastrointestinal, central nervous system, or vascular manifestations sometimes show a refractory clinical course, which affects the patient's prognosis. The gastrointestinal manifestation of BD, called intestinal BD, has been reported to be associated with morbidity and mortality.^[1]^

Several case reports of BD associated with myelodysplastic syndrome (MDS) have also been published. The BD patients complicated with MDS often show refractory disease with poor prognosis, especially in those accompanied by the cytogenetic abnormality trisomy 8. Such patients are refractory to immunosuppressive treatment, even if anti-tumor necrosis factor (TNF) inhibitors are used.^[2]^ However, some patients received hematopoietic stem cell transplantation (HSCT) to treat both BD and MDS, resulting in complete remission.^[3,4]^ Herein, we present the case of a patient with intestinal BD associated with trisomy 8-positive MDS who received abdominal surgery for ileocecal perforation followed by peripheral blood cell transplantation (PBSCT).

## 2 Case report

An 18-year-old woman visited our hospital with a 7-year history of BD for fever, oral aphthae, arthralgia, and genital ulcers. When she was 11 years old, she had been treated with oral colchicine as an initial treatment, but her symptoms continued with repeated aggravation and remission. Biologic agents such as etanercept, infliximab, adalimumab, and tocilizumab, in combination with methotrexate, were sequentially administered. However, these combination therapies did not result in significant improvement.

**Figure 1:**
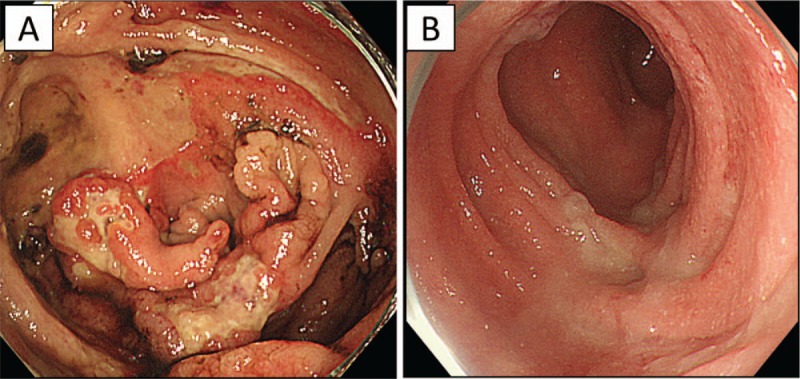
Colonoscopy findings before and after ileocecal resection. (A) Large, malformed, and widespread ulceration was seen in the cecum, with slight bleeding. (B) After ileocecal resection, there was no ulceration in ascending colonic mucosa.

**Figure 2:**
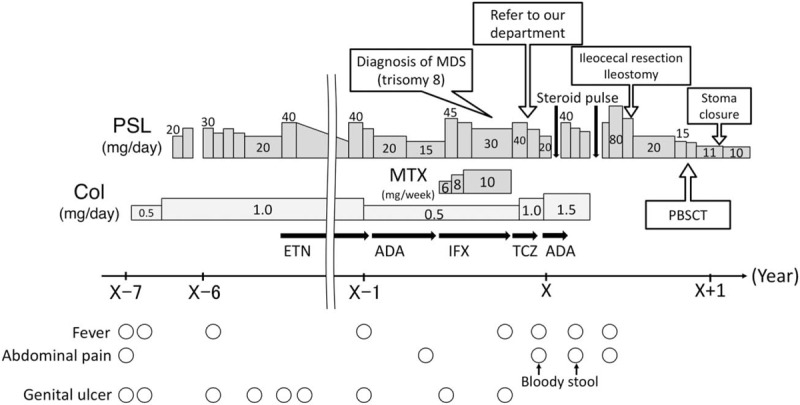
Clinical course of the present case. ADA = adalimumab, Col = colchicine, ETN = etanercept, IFX = infliximab, MDS = myelodysplastic syndrome, MTX = methotrexate, PBSCT = peripheral blood stem cell transplantation, PSL = prednisolone, TCZ = tocilizumab.

Physical examination revealed aphthae and mild tenderness in the lower right abdomen. Laboratory test results (Table 1) showed elevated levels of C-reactive protein and serum amyloid A. No elevation of monoclonal or polyclonal γ-globulin was found. The patient's human leukocyte antigen A26 was positive, but B51 was negative. Owing to a relatively low level of white blood cells despite a strong, persistent inflammatory condition, she was suspected of having some kind of immunodeficiency disease. To investigate any possible genetic abnormalities, chromosomal examination of peripheral blood was performed. The results indicated a karyotype of trisomy 8 in 87% of cells. Bone marrow examination showed dysplastic cells in multilineages, without atypical cells in peripheral blood. These findings were consistent with MDS/refractory cytopenia with multilineage dysplasia. The patient was thus referred to our department for further treatment, including HSCT. Colonoscopy revealed huge ulceration in the cecum and small erosions in the terminal ileum (Fig. 1A). Intravenous methylprednisolone was administered for the treatment of active intestinal BD and the oral prednisolone (PSL) dose was increased. Nevertheless, this did not alleviate her symptoms, especially intense abdominal pain. Thus, the patient subsequently underwent selective ileocecal resection and ileostomy because abdominal enhanced computed tomography showed thickening of the ileocecal wall with ascites around the cecum. The pathological findings in the resected specimen revealed that the cecum ulceration featured microperforation into the retroperitoneal space, forming small abscesses. The infiltrating cells were mainly neutrophils, and no abnormal cells were found. Colonoscopy after surgery showed no recurrence of intestinal ulcers (Fig. 1B). Six months after the surgical operation, the patient was admitted to the Department of Hematology in our hospital to receive allogeneic PBSCT with her mother as a donor. The transplantation was successful with no adverse events, resulting in the complete disappearance of trisomy 8 (Fig. 2). Seven months after the PBSCT, the symptoms of BD (fever, oral aphthae, abdominal pain, and genital ulcers) had disappeared and were kept in remission with oral PSL (10 mg/day).

**Table 1:**
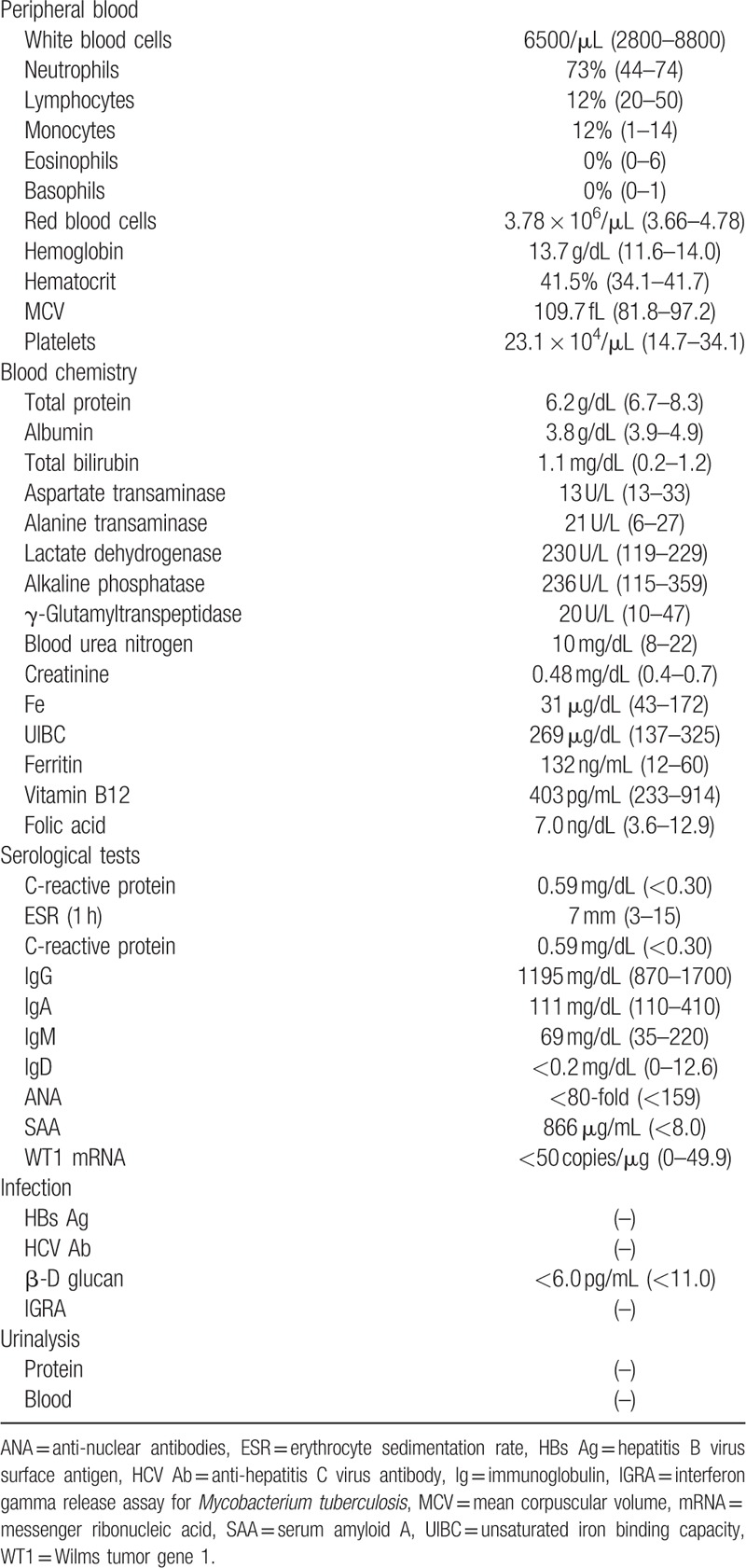
Laboratory findings on admission.

## 3 Discussion

The patient reported here showed refractory and severe intestinal BD symptoms and suffered intestinal perforation due to refractory ileocecal ulcers despite immunosuppressive therapies including biologic agents. After ileocecal resection, she received HSCT for refractory BD symptoms as well as MDS with trisomy 8. The HSCT procedure was successful and the PSL dose was successfully tapered with no relapse. To the best of our knowledge, this is the first case of intestinal BD with trisomy 8-positive MDS that received abdominal surgery and then HSCT, resulting in complete remission.

The association between intestinal BD and bone marrow failure (MDS and aplastic anemia) has been reported, predominantly from Asian countries.^[3–5]^ MDS is a prominent hematological complication of intestinal BD. For instance, Shen et al reported that about 2% of BD patients (16/805) were complicated with MDS in a Chinese cohort.^[5]^ Interestingly, intestinal BD patients with MDS are usually accompanied by a specific karyotype, trisomy 8.^[2,6]^ The rate of trisomy 8 positivity in intestinal BD patients associated with MDS was reported to be about 70%, in contrast to a lower frequency in patients with primary MDS (7%–9%).^[2,5,7,8]^ Intestinal BD with trisomy 8 is usually refractory.^[2,5,9]^

At present, there are no established treatments for intestinal BD, so previous experience with inflammatory bowel diseases such as Crohn disease or ulcerative colitis has often been used as basis for treatment.^[10]^ Immunosuppressive therapies, including biologic agents, can be used on refractory BD patients because TNF inhibitors are thought to be effective.^[11]^ Kimura et al reported that adalimumab treatment can be effective to control disease activity in patients with intestinal BD with trisomy 8-positive MDS.^[12]^ However, cases successfully treated using TNF inhibitors are very limited, indicating that these agents may not be effective enough.^[2,4,9]^ In fact, our case had received many different biologic agents, but they were not effective. The reason why BD patients with trisomy 8-positive MDS show a heightened inflammatory state is not fully understood; however, previous reports indicated that they have elevated levels of interleukin (IL)-1β, IL-6, IL-8, IL-17, IL-18, TNF-α, and interferon (IFN)-γ, especially in an active disease state.^[13,14]^ Similarly, Chen et al reported the upregulation of proinflammatory genes, such as *TGF-β* (transforming growth factor-β), *IFN-β2*, *IL-6*, *IL-7R*, *ICAM-1* (intercellular adhesion molecule-1), and *MCP-1* (monocyte chemotactic protein-1), in cluster of differentiation 34-positive hematopoietic progenitor cells from patients with MDS and trisomy 8.^[15]^ In the course of MDS progression, aberrant expression of inflammatory genes in blood cells may induce gastrointestinal ulcers or perforation, for example, the dysregulation of IL-7/IL-7 receptor signaling can initiate inflammatory colitis.^[16]^ In addition, increased IL-6 levels play an important role in the pathogenesis of inflammatory bowel disease.^[17]^ In our case, the complete remission of intestinal BD might be due to the reduction of expression of proinflammatory cytokine genes, for the complete disappearance of karyotype trisomy 8 in blood cells after HSCT.

The most effective therapy for BD with MDS may be HSCT.^[2,4]^ For example, Toyonaga et al reported that the only therapy with which both BD and MDS finally reached complete remission was HSCT.^[2]^Table 2 presents reported cases of intestinal BD with trisomy 8-positive bone marrow failure that received HSCT as curative treatment (10 cases including our case).^[3,4,9,18–21]^ The mean age of these patients was 29.1 years (4–64 years old) and most were from Asian countries. Only 1 patient was human leukocyte antigen-B51-positive. All patients showed intestinal ulcers, 90% of them had oral aphthae, and 60% had genital ulcers. In contrast, only 1 patient had eye involvement. In terms of the therapy, all described patients (for whom data were available) were administered glucocorticoid (6 of 6) and 2 patients each received colchicine, azathioprine, thalidomide, and infliximab. Ninety percent of patients were complicated with MDS (in 1 patient MDS transformed into acute myeloid leukemia M6). The prognosis after HSCT treatment appeared to be good; however, 3 patients died after it. The main cause of death was infection (enteritis in 1 case and pneumonia in 2 cases). Among the 10 cases, 2 received abdominal surgery for gastrointestinal complications (ileocecal fistula and perforation). Kawano et al reported that a 45-year-old male received abdominal surgery for ileocecal fistula into the ileum before HSCT. After this operation, the patient underwent PBSCT to treat both intestinal BD and MDS; however, he died of enteritis 3 months later.^[9]^

**Table 2:**
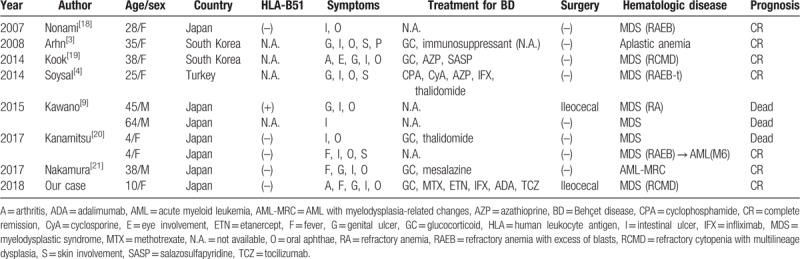
Clinical review of the patients with intestinal Behçet disease with trisomy 8-positive bone marrow failure that received hematopoietic stem cell transplantation as curative treatment.

These results indicate the apparent benefit of HSCT for curing both BD and hematological diseases; however, serious complications associated with immunosuppressive treatment should be considered.^[4]^ In other studies, azacytidine, an analog of the naturally occurring pyrimidine nucleoside cytidine, was shown to be effective in some patients with intestinal BD and trisomy 8-positive MDS.^[22,23]^ Therefore, for elderly patients in whom HSCT appears to have a high risk, an alternative therapeutic option such as azacytidine can be considered.

In conclusion, intestinal BD with trisomy 8-positive MDS can be refractory to immunosuppressive therapy. Therefore, HSCT is suggested for treating both conditions, especially in younger patients. In cases with severe gastrointestinal complications, aggressive abdominal surgery should be considered to stabilize the intestinal BD activity, after which curative HSCT therapy can then be performed. Further accumulation of such cases is needed to clarify the pathogenesis of intestinal BD with trisomy 8-positive MDS and to define an appropriate therapeutic strategy for this condition.

## Acknowledgments

The authors thank Edanz Group (www.edanzediting.com/ac) for editing a draft of this manuscript.

## Author contributions

**Conceptualization:** Tomoyuki Asano, Shuzo Sato, Kiyoshi Migita.

**Data curation:** Tomoyuki Asano.

**Formal analysis:** Tomoyuki Asano, Shuzo Sato.

**Investigation:** Tomoyuki Asano, Makiko Yashiro Furuya, Hiroshi Takahashi, Akiko Shichishima-Nakamura, Hiroshi Ohkawara, Tatsuo Fujiwara, Naohiko Gunji, Choichiro Hashimoto, Tomoyuki Momma, Motonobu Saito, Hiroshi Nakano, Guy Watanabe, Jumpei Temmoku, Yuya Fujita, Naoki Matsuoka, Mariko Mouri, Fumi Mashiyama, Hiroko Sakuma, Masaaki Mori.

**Methodology:** Tomoyuki Asano, Hiroshi Takahashi, Hiroko Kobayashi, Hiroshi Watanabe, Masaaki Mori, Takayuki Ikezoe, Kiyoshi Migita.

**Project administration:** Tomoyuki Asano, Kiyoshi Migita.

**Supervision:** Shuzo Sato, Hiroko Kobayashi, Hiromasa Ohira, Masaaki Mori, Takayuki Ikezoe, Kiyoshi Migita.

**Validation:** Shuzo Sato, Masaaki Mori, Takayuki Ikezoe, Kiyoshi Migita.

**Visualization:** Tomoyuki Asano, Hiroshi Watanabe.

**Writing – original draft:** Tomoyuki Asano, Shuzo Sato.

**Writing – review and editing:** Shuzo Sato, Kiyoshi Migita.
